# Captivity affects diversity, abundance, and functional pathways of gut microbiota in the northern grass lizard *Takydromus septentrionalis*

**DOI:** 10.1002/mbo3.1095

**Published:** 2020-07-14

**Authors:** Jin Zhou, Yu‐Tian Zhao, Ying‐Yu Dai, Yi‐Jin Jiang, Long‐Hui Lin, Hong Li, Peng Li, Yan‐Fu Qu, Xiang Ji

**Affiliations:** ^1^ Jiangsu Key Laboratory for Biodiversity and Biotechnology College of Life Sciences Nanjing Normal University Nanjing China; ^2^ Hangzhou Key Laboratory for Ecosystem Protection and Restoration College of Life and Environmental Sciences Hangzhou Normal University Hangzhou China

**Keywords:** captivity, gut microbiota, high‐throughput sequencing, northern grass lizard, *Takydromus septentrionalis*

## Abstract

Animals in captivity undergo a range of environmental changes from wild animals. An increasing number of studies show that captivity significantly affects the abundance and community structure of gut microbiota. The northern grass lizard (*Takydromus septentrionalis*) is an extensively studied lacertid lizard and has a distributional range covering the central and southeastern parts of China. Nonetheless, little is known about the gut microbiota of this species, which may play a certain role in nutrient and energy metabolism as well as immune homeostasis. Here, we examined the differences in the gut microbiota between two groups (wild and captive) of lizards through 16S rRNA sequencing using the Illumina HiSeq platform. The results demonstrated that the dominant microbial components in both groups consisted of Proteobacteria, Firmicutes, and Tenericutes. The two groups did not differ in the abundance of these three phyla. *Citrobacter* was the most dominant genus in wild lizards, while *Morganella* was the most dominant genus in captive lizards. Moreover, gene function predictions showed that genes at the KEGG pathway levels2 were more abundant in wild lizards than in captive lizards but, at the KEGG pathway levels1, the differences in gene abundances between wild and captive lizards were not significant. In summary, captivity exerted a significant impact on the gut microbial community structure and diversity in *T*.* septentrionalis*, and future work could usefully investigate the causes of these changes using a comparative approach.

## Graphical abstract

The dominant taxon with the abundance of >1% in gut microbiota was compared between wild and captive northern grass lizards (*Takydromus septentrionalis*). Significant differences were found between the two groups of lizards at all levels from class down to genus. Our results provide an inference that captivity can affect the species diversity and abundance of gut microbiota in lizards.

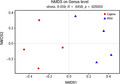

## 1 INTRODUCTION

The coevolutionary relationship between hosts and their gut microbiota has become a research hotspot in recent years. Gut microbiota plays an important role in energy budget (Semova et al., 2012), nutrient metabolism (Cani, 2016; Greer et al., 2016), immune homeostasis (Dimitriu et al., 2013), foraging behavior (Heijtz et al., 2011), and reproductive performance (Leftwich, Clarke, Hutchings, & Chapman, 2017). Numerous factors such as dietary structure, captivity, and host genetics can lead to changes in the gut microbial community structure (Gupta et al., 2012; Jiang et al., 2017; Kormas, Meziti, Mente, & Frentzos, 2014; Matsen, 2015; Rungrassamee et al., 2014). At present, the effects of ecological factors on the gut microbiota have been examined in a wide range of animal taxa, including mammals (Zhao et al., 2018), birds (Hird, Sánchez, Carstens, & Brumfield, 2015), reptiles (Zhang, Li, Tang, Liu, & Zhao, 2018), amphibians (Vences et al., 2016), fish (Sullam et al., 2012), and invertebrates (Rungrassamee et al., 2014). For instance, significant differences in the gut microbial community are discovered between wild crocodile lizards (*Shinisaurus crocodilurus*) and captive conspecifics fed with loaches (Jiang et al., 2017). Additionally, some similar bacterial characteristics are also found in the intestines between wild‐caught and domesticated black tiger shrimps (*Penaeus monodon*) (Rungrassamee et al., 2014). In brief, environmental and ecological factors take vital roles in the gut microbial community composition and abundance in animals (Sullam et al., 2012; Vences et al., 2016). However, further studies should be conducted to explore the impacts of these factors on the intestinal microbial characteristics, with the final goal of obtaining more general results on the relationship between hosts and their gut microbiota.

The structure and abundance of gut microbiota vary distinctly among different hosts, yet, the dominant gut microorganisms at the phylum level remain Firmicutes and Bacteroidetes in mammals, reptiles, and amphibians (Duncan et al., 2008; Kohl, Sadowska, Rudolf, Dearing, & Koteja, 2016; Vences et al., 2016; Zhang et al., 2018; Zhao et al., 2018), and Firmicutes and Proteobacteria in birds, fishes, and insects (Dewar, Arnould, Krause, Dann, & Smith, 2014; Li, Zhu, Yan, Ringø, & Yang, 2014; Wang, Cao, et al., 2016; Wang, Zheng, et al., 2016; Ye, Amberg, Chapman, Gaikowski, & Liu, 2014; Yun et al., 2014). Notably, Firmicutes can encode the energy metabolism‐related enzymes, have the potential to biosynthesize vitamin B, produce diverse kinds of digestive enzymes to break down various substances, and thus help their hosts digest and absorb nutrients (Flint, Scott, Duncan, Louis, & Forano, 2012; Rowland et al., 2018). On the other hand, the main function of Bacteroidetes is to ferment carbohydrates, degrade plant‐derived material, and short‐chain fatty acids, and thus improve the host's nutritional condition (Colston & Jackson, 2016). Additionally, Proteobacteria contribute to the cellulose activity, degrade a variety of aromatic compounds, and boost the nutrient absorption of their hosts (Reid, Addison, Macdonald, & Lloyd‐Jones, 2011). However, the abundance and composition of gut microbiota are affected by various factors, including geographical region, domestication, and genotype of hosts. In other words, the function of gut microbiota is mainly affected by the genetic background of hosts as well as the unique microhabitats (Suzuki & Worobey, 2014). In humans, for example, the abundance of Firmicutes is positively correlated with latitude, whereas the abundance of Bacteroidetes is negatively correlated with the latitude (Suzuki & Worobey, 2014). Moreover, the gut microbiota similarity shows a strong negative correlation with the genetic distance between hosts in the Adelie penguin *Pygoscelis adeliae* (Banks, Cary, & Hogg, 2009). Also, the different food resources in host microhabitats and captivity may affect the diversity and abundance of the gut microbiota in hosts.

Reptiles display immense diversities in their body size and shape, behavior, and life‐history strategies (Feldman, Sabath, Pyron, Mayrose, & Meiri, 2016; Shine, 2005; Woltering, 2012). These diversities will have an impact on the diversity and abundance of gut microorganisms. Numerous studies on reptiles have revealed that the gut microbiota is affected by the host habitats (Zhang et al., 2018), feeding habits (Campos, Guivernau, Prenafeta‐Boldú, & Cardona, 2018; Jiang et al., 2017), ontogeny (Price et al., 2017), captivity (Kohl et al., 2017), and adaptive radiation (Ren, Kahrl, Wu, & Cox, 2016), and that the gut microbiota also exerts a critical role in the health and development of their hosts (Berry et al., 2012; Dimitriu et al., 2013). For instance, juvenile green turtles (*Chelonia mydas*) acquire a polysaccharide fermenting gut microbiota quickly when they have settled into the coastal habitats of Brazil (Campos et al., 2018). It is necessary to investigate the gut microbial ecology of reptiles, especially for those using different diets and microhabitats, and additional basic experiments should be carried out on gut microbial ecology in reptiles, to understand the coevolutionary relationship between gut microbiota and their hosts.

The effects of environmental and ecological factors on the gut microbiota have been extensively investigated (Benson et al., 2010; Org et al., 2015; Spor, Koren, & Ley, 2011; Sullam et al., 2012; Yun et al., 2014; Zhang et al., 2018; Zhao et al., 2018). The impact of artificial perturbations and captivity on gut microbiota has been well investigated in Australian sea lions (*Neophoca cinerea*) (Delport, Power, Harcourt, Webster, & Tetu, 2016), southern elephant seals (*Mirounga leonine*) (Nelson, Rogers, Carlini, & Brown, 2013), leopard seals (*Hydrurga leptonyx*) (Nelson et al., 2013), deer mice (*Peromyscus maniculatus*) (Schmidt, Mykytczuk, & Schultehostedde, 2019), several species of monkeys (Hale et al., 2018; Nakamura et al., 2011; Villers, Jang, Lent, Lewin‐Koh, & Norosoarinaivo, 2008), Kinda baboons (Tsukayama et al., 2018), anteaters (Delsuc et al., 2014), woodrats (Dewar et al., 2014), and lizards (Colston, 2017). The results suggest that animals exposed to human activities and/or held in captivity may change gut microbial compositions and abundances. Such changes may lead to intestine‐related dysfunction or even disease in the hosts, like inflammatory bowel disease (IBD) (Berry et al., 2012), Crohn's disease (Opstelten et al., 2016), and inflammation (Boulange, Neves, Chilloux, Nicholson, & Dumas, 2016). Meanwhile, certain changes or selective manipulation of gut microbial compositions and abundances may also improve the immune status of hosts (Montalban‐Arques et al., 2015) and even favorably affect host development and behavior (Heijtz et al., 2011). Raising animals in captivity or feeding them with different types of food can alter their gut microbial structure and abundance. Nonetheless, only a few studies have been performed to examine the differences in gut microbial compositions and abundances between wild and captive populations, and these studies involve lizards (Colston, 2017; Nelson, Cann, Altermann, & Mackie, 2010), birds (Dewar et al., 2014; Hird, 2017), and mammals (Delport et al., 2016; Delsuc et al., 2014; Hale et al., 2018; Nakamura et al., 2011; Schmidt et al., 2019; Tsukayama et al., 2018; Villers et al., 2008).

Taken together, the diversity and abundance of gut microbiota are related to the integration of strong natural selection, coevolution between microbiota and hosts, and host habitats (Blaut et al., 2002; Magne et al., 2006). In this process, the host provides a living environment and adequate food resources for gut microbiota, while the latter assists the host in decomposing and digesting substances, thus providing more nutrients for the host (Magne et al., 2006). For instance, the herbivorous tetrapods have to build the endosymbiotic relationships between hosts and microbes, since they cannot express hydrolases for cellulose and hemicellulose (Campos et al., 2018). In this regard, the mutually beneficial relationship between hosts and their gut microbiota has been certified to maintain host homeostasis, including the regulation of the immune system and metabolism‐related functions (Suzuki & Worobey, 2014; Zhang et al., 2018).

The northern grass lizard (*Takydromus septentrionalis*) is a small‐sized, multiple‐clutched oviparous lacertid lizard endemic to China and has a range covering the central and southeastern parts of the country (Liu, 1999). The lizard consists of three divergent lineages, with isolation by a distance known to be the main cause of genetic divergence (Cai, Yan, Xu, Lin, & Ji, 2012). Spatio‐temporal variation in life‐history (e.g., size at maturation, adult size, clutch size, clutch frequency, and egg size; Du, Ji, Zhang, Xu, & Shine, 2005; Du, Ji, Zhang, Lin, & Xu, 2010; Ji & Diong, 2006; Ji, Du, Lin, & Luo, 2007) and physiological (e.g., thermal preference, thermal tolerance and thermal dependence of food assimilation, energy allocation, and locomotor performance; Ji, Du, & Sun, 1996; Luo, Ding, & Ji, 2010; Yang, Sun, An, & Ji, 2008) traits is evident for this lizard, with proximate factors being less important determinants of such variation than are genetic influences. In this study, we used a sample of 23 adult males to examine the differences in gut microbial composition between the wild and captive individuals through MiSeq sequencing of bacterial 16S rDNA from the samples, to examine whether domestication had a significant influence on gut microbial composition, abundance, and function.

## 2 MATERIALS AND METHODS

### 2.1 Lizard collection and maintenance

Twenty‐three adult males were collected using a noose in mid‐April 2019 from Tangshan (32°3′N, 119°1′E), Nanjing, Jiangsu, eastern China. Snout–vent lengths (SVLs) ranged from 56.4–83.8 mm, with a mean of 65.6 mm; body masses ranged from 3.8–11.1 g, with a mean of 5.9 g. Great efforts were made to ensure that all individuals we collected were healthy and free of ectozoic parasites. Only adult males were used in this study, thereby removing the possible influence of gender. The 23 lizards were randomly divided into two groups, thereby minimizing the possible influence of size or age. One group (*N* = 11) was used to extract and amplify the DNA of gut microbiota. Briefly, these 11 lizards were euthanized immediately through lethal injection of MS‐222 for further processing. The other group (*N* = 12) was transported to our laboratory in Nanjing, where lizards were raised under the same conditions for 90 days from mid‐April to mid‐July. More specifically, these lizards were individually maintained in 640 × 450 × 380 mm (length × width × height) plastic containers with soil sterilized by autoclave and fed with a 1/1 (mass/mass) mixture of mealworms (*Tenebrio molitor*) and crickets (*Achetus domesticus*) and distilled water supplemented with the vitamin and minerals. The diet was sterilized by ultraviolet irradiation and water by autoclaving before feeding to the lizards. The plastic contains were placed in an outdoor enclosure to simulate the natural conditions. The captive lizards were euthanized in mid‐July using the method described above. Our experimental procedures were approved by the Institutional Animal Care and Use Committee of Nanjing Normal University and were conducted following related guidelines.

### 2.2 DNA extraction and amplification

The full intestinal tract was collected, squeezed, and scraped to collect adequate contents under sterile conditions. Over 250 mg contents should be collected from every tube; as a result, the gut contents were mixed with 2–3 individuals and transferred into a sterile centrifuge tube. Then, the sterile centrifuge tube was frozen at −80°C after weighing, until DNA was extracted from them.

The E.Z.N.A.® stool DNA Kit (Omega Bio‐tek) was used to extract the total genomic DNA following the manufacturer protocols. The quantity and quality of the extracted DNA were measured using Qubit@ 2.0 Fluorometer (Thermo Scientific) and 1.0% agarose gel electrophoresis, respectively. Thereafter, the 16S rRNA V3‐V4 genes were amplified through the universal bacterial primers 341F and 805R. Moreover, polymerase chain reaction (PCR) was performed in the reaction system with the total volume of 30 μl, which was supplemented with 2 μl DNA primer, 15 μl of 2 × Taq master Mix, and 20 ng genomic DNA. Further, the PCR thermal cycling conditions were as follows: initial denaturation at 94°C for 3 min, followed by 5 cycles of denaturation at 94°C for 30 s, annealing at 45°C for 20 s and extension at 65°C for 30 s. Also, the other 20 cycles consisted of 94°C for 20 s, 55°C for 20 s, and 72°C for 30 s, with a final extension at 72°C for 5 min. At the second stage of the two‐step PCR process, the 8‐base barcodes were introduced for multiplex sequencing. In the meantime, the 5‐cycle PCRs were conducted to incorporate the two unique barcodes into either end of the 16S rDNA amplicons. The thermal cycling conditions were as follows: denaturation at 95°C for 3 min, followed by 5 cycles of 94°C for 20 s, 55°C for 20 s, and 72°C for 30 s, and the final extension at 72°C for 5 min. Afterward, the PCR products were purified using the MagicPure Size Selection DNA kit (Transgen) and quantified using the Qubit dsDNA HS assay kit (Invitrogen, American). For further analysis, equivalent amounts of PCR amplicons were sequenced on the MiSeq platform using the MiSeq 3 Reagent kit (Illumina, Sangon Biotech Co., Ltd).

### 2.3 Sequence and data analysis

After trimming the primer sequence, PEAR 0.9.6 was adopted to merge the paired‐end reads from the original DNA fragments (Zhang, Kobert, Flouri, & Stamatakis, 2014). Then, these joined sequences were assigned to each sample based on their unique barcodes, and filtered according to the base quality using PRINSEQ 0.20.4 (Schmieder & Edwards, 2011). Subsequently, chimeric sequences were identified and removed by UCHIME 4.2.40 (Edgar, Haas, Clemente, Quince, & Knight, 2011), and the resultant sequences were deposited in the Sequence Read Archive database of NCBI (Accession No. PRJNA597659).

Afterward, the available sequences were clustered into operational taxonomic units (OTUs) according to the similarity threshold of 97% through USEARCH 5.2.236 (Edgar, 2010). The representative sequence from each OTU was aligned with the 16S rRNA Greengenes 13.5 database, to annotate the taxonomic information, and the classified confidence was set at 70%. Later, the rarefaction curve and alpha diversity analyses, namely the community richness parameters (Chao1 index), the community diversity parameters (Shannon index), the Good's coverage, and abundance‐based coverage estimator (ACE), were calculated through MOTHUR 1.30.1 (Schloss et al., 2009) and visualized by R 3.6 (R Development Core Team, 2019).

For beta‐diversity analysis, the non‐metric multidimensional scaling (NMDS) was constructed based on the Bray–Curtis through the vegan package in R (Oksanen et al., 2013). Besides, the linear discriminant analysis effect size (LEfSe) (Segata et al., 2011) was adopted for constructing the linear discriminant analysis (LDA), which allowed for searching for the taxon with significantly different relative abundance between wild and captive lizards. Besides, the differences in intestinal microbial composition between wild and captive lizards were analyzed using a *t* test.

Further, PICRUSt (Langille et al., 2013) was used to predict all OTUs in the intestinal microbiota of lizards based on the Kyoto Encyclopedia of Genes and Genomes (KEGG) database (Kanehisa, 2019). Then, the PICRUSt results were analyzed and compared using the Student's *t* test, and the confidence intervals (CIs) were set to 0.95. All values were presented as mean ±*SE*. All statistical analyses were conducted at the significance level of *α* = 0.05.

## 3 RESULTS

### 3.1 Gut bacterial sequencing

**FIGURE A1:**
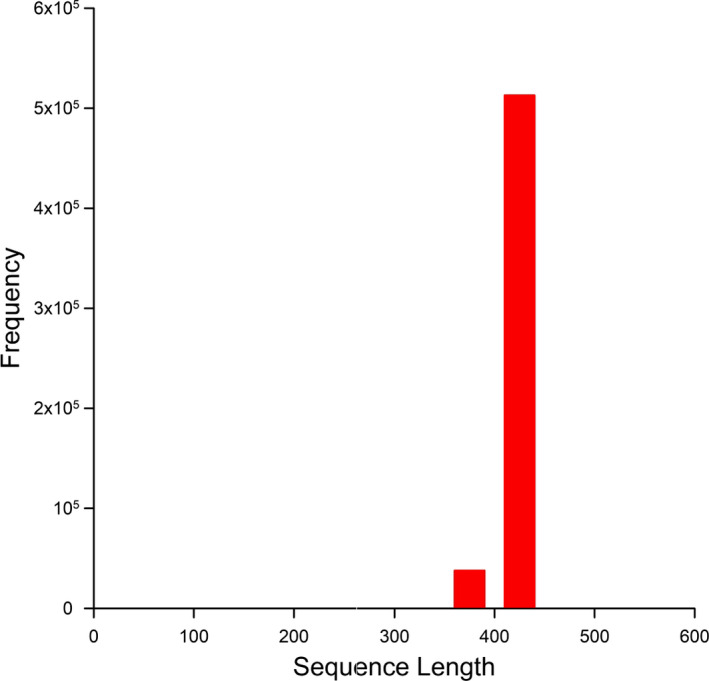
Length distribution of trimmed sequences

**FIGURE A2:**
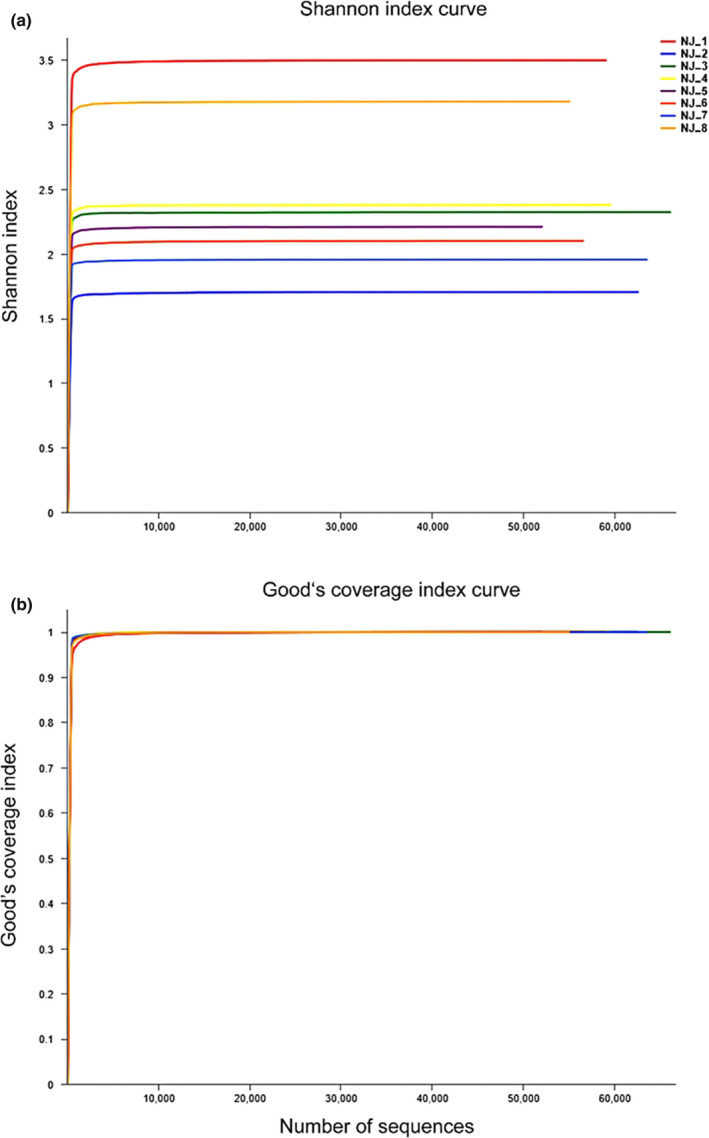
Alpha diversity index curve. (a) Shannon index curve; (b) Good's coverage index curve

A total of 289,995 and 304,289 raw reads were obtained from wild and captive lizards, respectively. After quality filtering, altogether 226,289 and 246,508 high‐quality reads were obtained, with an average sequence length of 420 bp (range, 351–462 bp) (Figure A1). Specifically, an average of 56,572 ± 2,056 reads (range, 51,888–63,116 reads per sample) were obtained from the 4 intestinal samples from wild lizards, while an average of 61,627 ± 1,439 reads (range, 58,820–66,049 reads per sample) were acquired from the 4 intestinal samples from captive lizards. Moreover, the total number of OTUs at the 97% similarity level was 286 for the lizards used in this study (average, 133 ± 12 OTUs per sample), including 237 (range, 96–208) in wild lizards (average, 137 ± 4 OTUs per sample), and 256 (range, 91–208) in captive lizards (average, 129 ± 12 OTUs per sample). The Shannon–Wiener index curve for all samples showed that a sufficient amount of OTUs was detected and leveled off generally at this sequencing depth, suggesting that there were sufficient sequences for further analyses (Figure A2). Further, the Good's coverage estimation minimum values were >99.9%, which indicated that most gut bacterial communities of diverse species were retrieved from these samples (Figure A2).

### 3.2 Gut microbiota composition

**FIGURE 1:**
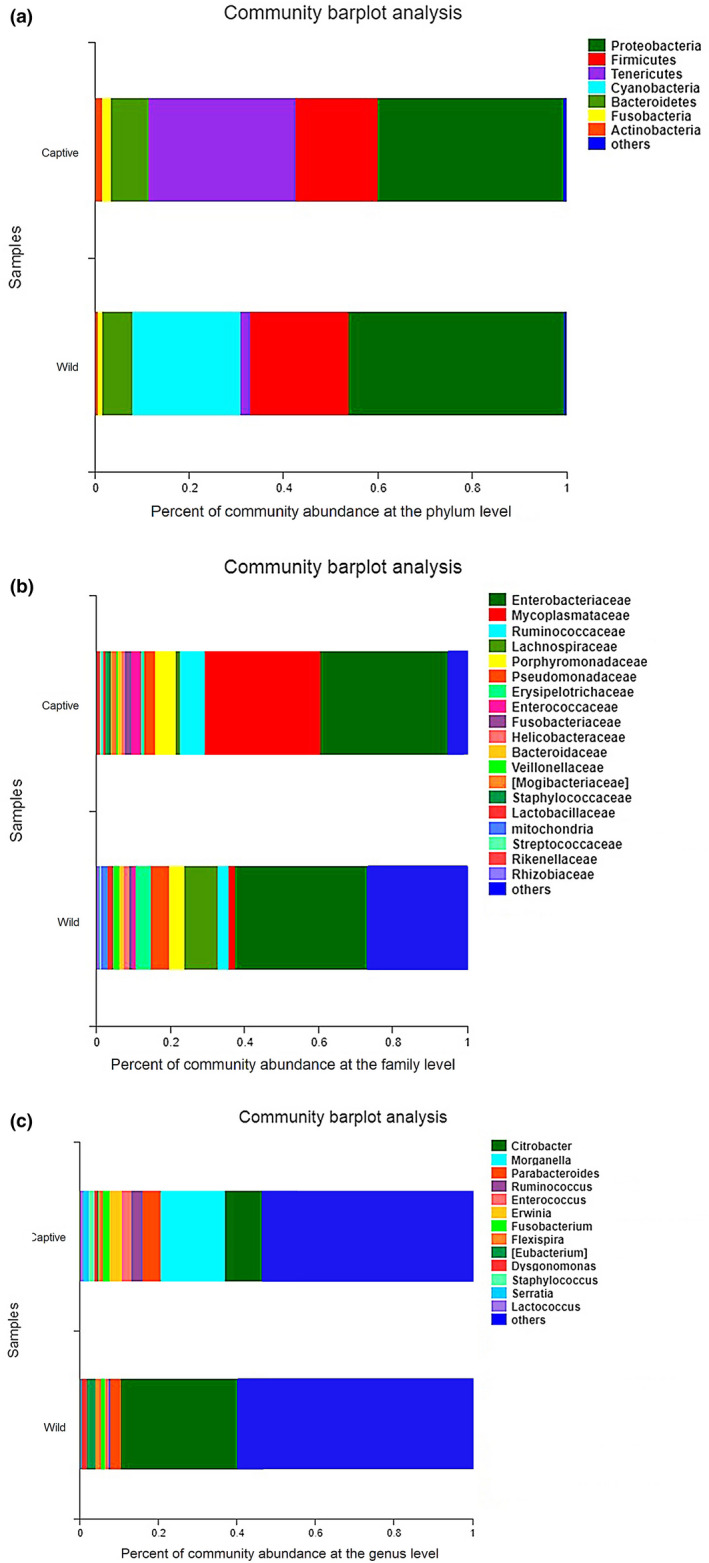
The relative abundance of intestinal microbiota between wild and captive lizards at the phylum (a), family (b), and genus (c) levels. Only phyla, family, or genus with relative abundance greater than 1% are shown in the histogram and the other taxons are combined

On the whole, representatives of 12 known microbial phyla, 26 microbial classes, 46 microbial orders, 99 microbial families, and 154 microbial genera were detected in *T*.* septentrionalis* based on taxonomic assignment at the sequencing identity level of 97% (Table A1). Also, representatives from 12 phyla, 24 classes, 42 orders, 92 families, and 142 genera were detected in the intestinal samples from captive lizards at the sequence identity level of 97%. Similarly, in wild lizards, representatives of 12 phyla, 26 classes, 43 orders, 92 families, and 143 genera were discovered in the intestinal samples. Figure 1 shows the proportions of microbiota under different taxonomic classifications. At the phylum level, the intact gut microbiota was mainly dominated by Proteobacteria (42.89 ± 7.11%), Firmicutes (19.25 ± 2.76%), and Tenericutes (16.53 ± 8.17%), while other representative phyla included Cyanobacteria (10.86 ± 6.64%), Bacteroidetes (7.51 ± 4.53%), Fusobacteria (1.40 ± 0.57%), Actinobacteria (1.18 ± 0.51%), and Deferribacteres (0.31 ± 0.16%), which accounted for about 99.93% (Figure 1a). As for captive lizards, Proteobacteria (39.02 ± 8.45%), Tenericutes (31.09 ± 12.64%), and Firmicutes (17.49 ± 4.99%) (Figure 1a) were the dominant phyla in the gut microbiota. Compared with the captive group, wild lizards were rich in Proteobacteria (46.77 ± 11.10%), Cyanobacteria (21.70 ± 10.85%), and Firmicutes (21.01 ± 1.98%), which occupied over 89.47% (Figure 1a).

At the family level, Enterobacteriaceae (34.98 ± 7.61%) and Mycoplasmataceae (16.48 ± 8.15%) were dominant in the gut microbiota, whereas other families with quantitative advantages included Ruminococcaceae (4.97 ± 1.93%), Lachnospiraceae (4.96 ± 1.64%), Porphyromonadaceae (4.96 ± 3.08%), Pseudomonadaceae (4.10 ± 2.58%), Erysipelotrichaceae (2.54 ± 1.26%), Enterococcaceae (1.69 ± 0.53%),Fusobacteriaceae (1.39 ± 0.58%), Helicobacteraceae (1.31 ± 0.62%), and Bacteroidaceae (1.03 ± 0.56%) (Figure 1b). In captive lizards, the dominant families were Enterobacteriaceae (33.70 ± 10.36%) and Mycoplasmataceae (31.00 ± 12.61%), with the pooled relative abundance of over 64.70% (Figure 1b). However, the dominant family in wild lizards only included Enterobacteriaceae (36.25 ± 11.11%), and no other family accounted for >9% (Figure 1b).

At the genus level, the most dominant genus was *Citrobacter* (19.70 ± 6.45%), while other predominant genera included *Morganella* (7.96 ± 5.08%) and *Parabacteroides* (3.76 ± 2.39%) (Figure 1c). In captive lizards, the most dominant genus was *Morganella* (15.90 ± 8.47%), whereas genera with a relative abundance of >3% included *Citrobacter* (9.37 ± 1.84%) and *Parabacteroides* (4.69 ± 4.06%) (Figure 1). Nonetheless, the dominant genus in wild lizards only included *Citrobacter* (30.04 ± 10.47%), while other identifiable genera had a relative abundance of <3%, including *Parabacteroides* (2.83 ± 2.44%), *Eubacterium* (2.29 ± 1.28%), and *Flexispira* (1.57 ± 1.17%) (Figure 1c).

### 3.3 Effects of captivity on the gut microbiota

The alpha diversities, including the Shannon diversity index, the Simpson diversity index, Chao1 richness, the abundance‐based coverage estimator (ACE), and the Good's coverage index, were employed to evaluate the diversity differences in gut microbial community between wild and captive lizards (Table 1). No differences were detected in Shannon, Simpson, Chao1, and ACE (all *p* > 0.45) upon Student's *t* test, except for Good's coverage (*t* = 3.06, *df* = 6, *p* = 0.02).

**TABLE 1:** The summary of diversity indexes and comparing with Student's *t* test between wild and captive lizards

Diversity indexes	Captive group	Wild group	Student's *t* test results
Shannon	2.47567 ± 0.32337	2.36011 ± 0.240370	*t* = 0.25, *df* = 6, *p* = 0.81
Simpson	0.21660 ± 0.06419	0.18954 ± 0.032602	*t* = 0.33, *df* = 6, *p* = 0.76
Ace	152.00427 ± 20.17658	174.28415 ± 12.74231	*t* = 0.81, *df* = 6, *p* = 0.45
Chao 1	144.58482 ± 22.43495	164.30238 ± 9.18874	*t* = 0.70, *df* = 6, *p* = 0.51
Good's coverage	0.99969 ± 0.00001	0.99951 ± 0.00005	*t* = 3.06, *df* = 6, *p* = 0.02

Specifically, the Student's *t* test was carried out to examine the differences in intestinal microbiota with an abundance of >1% between wild and captive lizards, and the results indicated significant differences between the two groups at class, order, family and genus levels. Nonetheless, there was no significant difference at the phylum level (all *p* > 0.09); thus, these patterns were not discussed further in this study. Meanwhile, abundances of Alphaproteobacteria (*t* = 2.64, *df* = 6, *p* = 0.038) and Betaproteobacteria (*t* = 4.02, *df* = 6, *p* = 0.007) at the class level, Bacillales (*t* = 3.00, *df* = 6, *p* = 0.024) at the order level, Lachnospiraceae (*t* = 3.93, *df* = 6, *p* = 0.008), Veillonellaceae (*t* = 3.40, *df* = 6, *p* = 0.015), and Staphylococcaceae (*t* = 2.72, *df* = 6, *p* = 0.035) at the family level differed significantly between wild and captive lizards. More specifically, wild lizards had a higher proportion of Alphaproteobacteria at the class level, as well as Lachnospiraceae and Veillonellaceae at the family level. By contrast, captive lizards had a higher percentage of Betaproteobacteria at the class level, Bacillales at the order level, and Staphylococcaceae at the family level (Table A2).

**FIGURE 2:**
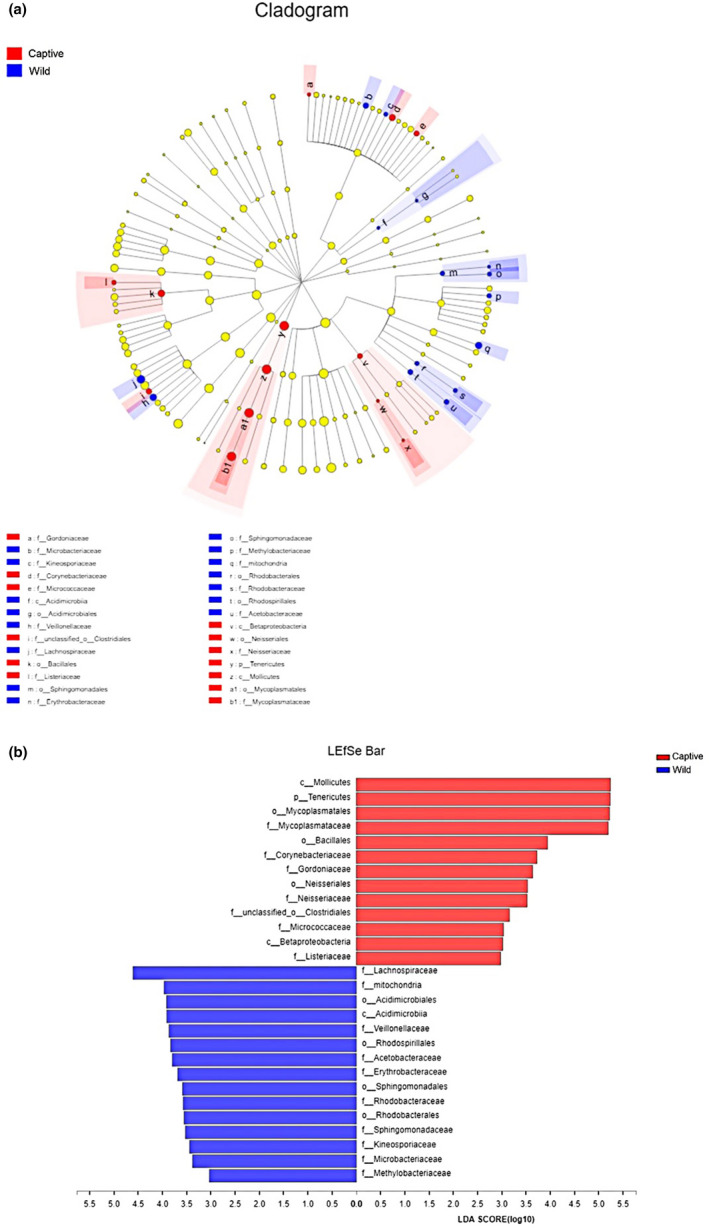
Linear discriminant analysis effect size (LEfSe) analysis of gut microbiota composition between wild and captive lizards (LDA > 2.9, *p* < 0.05). (a) Taxonomic representation of statistically and biologically consistent differences between wild and captive lizards. Differences are represented using a colored circle, color in circles represent their respective levels of classification, and circle size is proportional to the taxon's abundance, represents the Phylum, the class, the order, and the family. (b) Histogram of the LDA scores computed for features differentially abundant between wild and captive lizards. LEfSe scores can be interpreted as the degree of consistent difference in the relative abundance of analyzed microbial communities between wild and captive lizards

Further, LEfSe analyses were performed on samples to estimate the difference in relative abundance (averages of relative abundance >1%) at different bacterial taxonomic levels (including phylum, class, order, and family). The results suggested that a greater proportion of Mollicutes at the class level (*LDA* = 5.49, *p* = 0.04), Tenericutes at the phylum level (*LDA* = 5.24, *p* = 0.04), Mycoplasmatales at the order level (*LDA* = 5.22, *p* = 0.04), and Mycoplasmataceae at the family level (*LDA* = 5.19, *p* = 0.04) were found in captive lizards (Figure 2).

**FIGURE 3:**
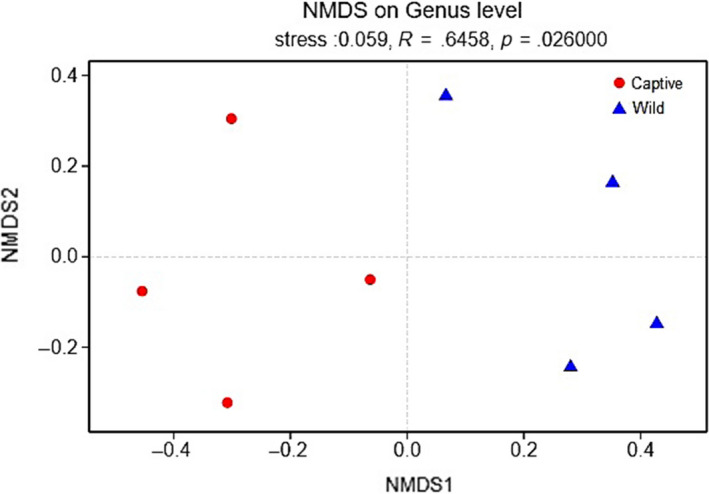
NMDS ordination based on Bray–Curtis similarities of bacterial communities between wild and captive lizards

Also, NMDS was conducted to analyze the beta‐diversity for wild and captive lizards at the genus level. Results of similarity analysis indicated a significant difference in intestinal microbiota between wild and captive lizards (ANOSIM test, Stress: 0.056, *R* = 0.65, *p* = 0.026; Figure 3).

### 3.4 The predicted metagenomes

**FIGURE 4:**
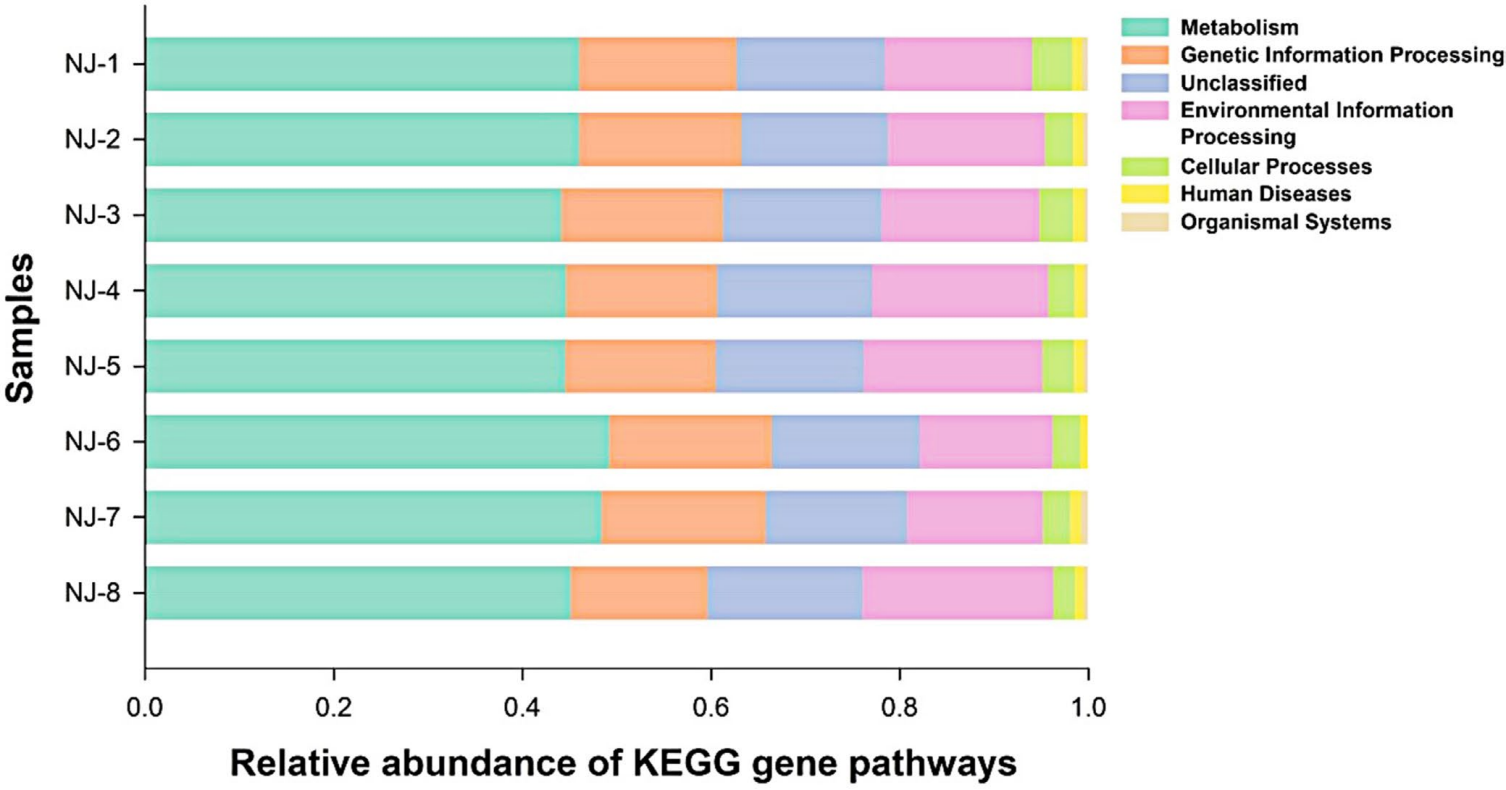
Relative abundance of gut bacterial taxa at KEGG gene pathways

To better understand the functional differences between wild and captive lizards, PICRUSt was used as a macro‐genome inference method for the 16S rRNA dataset, with the average weighted NSTI values of 0.07 ± 0.01 and 0.16 ± 0.04 for wild and captive lizards, respectively. A total of 5,386 KEGG genes were obtained from the whole dataset, among which, 5,148 were detected in both groups. To understand the changes in functional compositions, all KEGG genes were further mapped to the KEGG categories and pathways. Results of Student's *t* test revealed no statistically significant functional difference in gut microbiota between wild and captive lizards at the KEGG pathway levels1 (all *p* > 0.23). Notably, a majority of the resultant KEGG categories belonged to metabolism (45.99 ± 0.62%), environmental information processing (16.92 ± 0.74%), genetic information processing (16.51 ± 0.33%), cellular processes (3.10 ± 0.20%), human diseases (1.09 ± 0.03%), and organismal systems (0.59 ± 0.05%) (Figure 4, Table A2). Furthermore, these KEGG categories were assigned to 35 different subcategories, and the KEGG abundances at pathways levels2 had significantly higher values in wild lizards than those in captive lizards (all *p* < 0.0394) except for the circulatory system belonging to the organismal systems (*t* = 2.25, *df* = 6, *p* = 0.065) (Table A2).

## 4 DISCUSSION

It has been reported in some studies that captivity cannot change the gut microbiota (Muegge et al., 2011; Rungrassamee et al., 2014). Nonetheless, our results revealed that captivity affected the gut microbiota in *T*.* septentrionalis*. Such a discrepancy in results might be ascribed to differences in diets and environments between studies (Delsuc et al., 2014). Notably, differences in food resources represent the most direct and important factor affecting the diversity and abundance of gut microbiota (Wong et al., 2015; Yun et al., 2014). This is especially true for species with different feeding habits at different developmental stages or in different microhabitats (Vences et al., 2016; Zhang et al., 2018; Zhao et al., 2018). For instance, the gut microbial community in loach‐fed crocodile lizards is distinctly different from that in the earthworm‐fed and wild lizards (Jiang et al., 2017), and the β‐diversity of gut microbiota in pika is related to diet diversity (Li et al., 2016). The increased fibers modulate the expression of numerous microbial metabolic pathways, such as glycan metabolism, and genes encoding the carbohydrate‐active enzymes are also active on fibers or host glycans in humans (Tap et al., 2015). These effects may be particularly pronounced in captive animals, and their diets are markedly different from those in wild animals (Delsuc et al., 2014; Ng, Stat, Bunce, & Simmons, 2018). Thus, the gut microbiota of dietary specialists may be more susceptible to captivity (Kohl, Skopec, & Dearing, 2014). Also, some of the factors that have important effects on gut microbiota might be directly or indirectly caused by diet. For instance, seasonal changes in diet composition can explain 25% of seasonal variation in microbiota composition across herbivorous mammals (Kartzinel, Hsing, Musili, Brown, & Pringle, 2019).

A large proportion of intestinal microbes in animals derive from local microorganisms, particularly those in soil, which significantly affect the population structure and abundance of intestinal microorganisms (Delsuc et al., 2014; Kohl et al., 2017). For example, the gut microbiota in giant armadillos (*Priodontes maximus*) is very similar to the soil where the microorganisms live, and this may be because part of prey items contains the associated soil microbiota (Delsuc et al., 2014; Vaz, Santori, Jansen, Delciellos, & D'Andrea, 2012). Besides, animals may also consciously ingest soil components to facilitate the ingestion of related substances, like chitin (Delsuc et al., 2014). Thus, the soil contains a dynamic reservoir of biodiversity, which plays a key role in sustaining and altering the diversities and abundances of gut microbiota in animals.

In this study, captive lizards showed higher gut microbial α‐diversity values than did wild lizards. Such a result is consistent with that reported for rhinoceros (Mckenzie et al., 2017). However, in other studies on canids, primates, equids, and woodrats, wild animals exhibit higher gut microbial α‐diversity levels than do captive conspecifics (Kohl et al., 2014; Mckenzie et al., 2017). Additionally, the gut microbial α‐diversity values are consistent in some studies on bovids, giraffes, anteaters, and aardvarks between wild and captive individuals (Mckenzie et al., 2017). Also, differences in gut microbial β‐diversity between the captive and wild groups were observed in most of the taxa surveyed. McKenzie et al. (2017) discovered that there were significant differences in gut microbial β‐diversity in mammals investigated, except for bovids and giraffes. Besides, LEfSe analysis and NDMS revealed significant differences between wild and captive lizards (Figures 2 and 3).

In the captive environment, the human‐constructed facilities (such as breeding, zoo, and simulated environments) have replaced the extreme conditions in the wild environment where animals live. Captive animals will undergo great changes in the gut microbiota structure and abundance. Most of these changes are attributed to the alterations in an environment where animals live, such as changes in foods, antibody, and veterinary medicine intervention, limited range of activity, reduced exposure to diverse habitat types and other species, and increased exposure to human‐related microbes and microbes that thrive in a captive environment, even though little research is carried out (Hyde et al., 2016; Mckenzie et al., 2017). In this study, lizards were raised in an environment similar to their wild habitats and were fed with mealworms, crickets, and water enriched with various vitamins and minerals for 3 months. Nonetheless, the causes of differences in the gut microbiota between wild and captive lizards were unknown, since environmental microbes were not collected and the food items were not controlled. Thus, more studies are warranted to draw further conclusions.

The dominant phyla of gut microbes found in this study included Proteobacteria, Firmicutes, and Tenericutes that did not differ between wild and captive lizards (Figure 1a). Furthermore, the composition of gut microbiota was unique, among which, the dominant phyla were Proteobacteria, Tenericutes, and Firmicutes in captive lizards, and were Proteobacteria, Cyanobacteria, and Firmicutes in wild lizards (Figure 1a). The relative abundance of gut microbes varies significantly among reptile species. In other lizards, the dominant phyla of gut microbes are Firmicutes (33.2%–73.0%), Bacteroidetes (6.2%–45.6%), and Proteobacteria (5.7%–62.3%) (Hong, Wheeler, Cann, & Mackie, 2011; Jiang et al., 2017; Kohl et al., 2017; Martin, Gilman, & Weiss, 2010; Nelson et al., 2010; Ren et al., 2016; Zhang et al., 2018). In our study, the difference in the phylum Tenericutes was statistically significant between wild and captive lizards according to LEfSe analyses (Figure 2).

Proteobacteria may be the third most abundant phylum in mammalian gut microbiota, which has been recognized as the dominant phylum in some fish (Wong et al., 2015), reptiles (Jiang et al., 2017), and birds (Colston & Jackson, 2016; Hird et al., 2015). Proteobacteria are associated with diverse metabolism and typical decomposition, fermentation of complex sugars, and vitamin production (Colston & Jackson, 2016). Meanwhile, Firmicutes can encode enzymes involved in digestion and produce a variety of digestive enzymes for degrading the nutrient substances, thus assisting their hosts in nutrient digestion and absorption (Colston & Jackson, 2016; Hale et al., 2018). As for the vertebrate gut microbiota, the Tenericutes members have been identified as the important members of gut communities in fish, amphibians, reptiles, and mammal, which may exert certain roles in nutrient processing (Colston & Jackson, 2016). Tenericutes may also be related to patients with metabolic syndrome, which is suggested as a heritable taxon in humans (Lindheim et al., 2017). In our study, captive lizards had a higher proportion of Tenericutes, which might be related to the limited activity space and food sources of lizards. Inversely, some Cyanobacteria members may be protected by their mucilaginous coverings and subcultured through the gut in alive hosts (Lewin, Kamjunke, & Mehner, 2003). The wild lizards ingest insects that feed on Cyanobacteria microorganisms, which may account for why many Cyanobacteria members are found in some samples.

Nonetheless, few existing studies have discussed the composition and abundance of intestinal microbiota at the class and/or order levels. In this study, wild lizards had a higher abundance of Alphaproteobacteria bacteria than captive lizards at the class level. Moreover, captive lizards had a higher abundance of Betaproteobacteria bacteria at the class level, as well as Bacillales bacteria at the order level than did captive lizards. Alphaproteobacteria and Betaproteobacteria belong to Alphaproteobacteria, and they only contribute to a few enzymes (Nechitaylo et al., 2010). Moreover, the functions of Bacillales may be related to carbohydrate metabolism (Do et al., 2014). According to our results, a total of 28 characteristic taxa (from phylum to genus) associated with wild and captive groups were identified through LEfSe analyses, with the LDA threshold of ≥3.0 (Figure 2). Differences in gut microbiota at the class (3/28) and order (8/28) levels were statistically significant between wild and captive groups (Figure 2). At the class level, Betaproteobacteria (phylum Proteobacteria) and Mollicutes (phylum Tenericutes) were more abundant in captive lizards, while Acidimicrobiia (phylum Actinobacteria) was more abundant in wild lizards. At the order level, some belonged to classes Alphaproteobacteria (3/8) and Betaproteobacteria (1/8), and others included Acidimicrobiales, Bacillales, Clostridiales, and Mycoplasmatales (Figure 2).

The dominant microbial families in *T*.* septentrionalis* were Enterobacteriaceae of the phylum Proteobacteria and Mycoplasmataceae of the phylum Tenericutes. Further, Enterobacteriaceae was predominant in both wild and captive lizards, while the subdominant family Mycoplasmataceae was more abundant in captive lizards. Differences in the gut microbiotas between wild and captive lizards might be attributed to the changes in the relative abundances of particular microbial families. Sixteen characteristic families associated with wild and captive lizards were identified according to LEfSe analyses with the LDA threshold of ≥2.9 (Figure 2). These families were primarily subordinate to the phyla Proteobacteria (7/16), Actinobacteria (5/16), Firmicutes (3/16), and Tenericutes (1/16). Further, the abundance of Staphylococcaceae was dramatically higher in captivity than in wild lizards; thus, the abundances of Lachnospiraceae and Veillonellaceae were lower in captivity than in wild lizards. At the genus level, the most dominant genus was *Citrobacter* in wild lizards and *Morganella* in captive lizards. However, these genus‐level differences were not statistically significant (Figures 1, 2, 3).

Captivity affected the microbial community structure and species richness of intestinal microbiota. Results of Student's *t* test and LEfSe analysis indicated significant differences in species richness between wild and captive lizards (Table 1; Figures 1, 2, 3). NDMS also suggested significant differences in intestinal microbial community structure between wild and captive lizards (Table 1; Figures 1, 2, 3). These findings indicate that captivity might result in the significantly altered intestinal microbiota structure and abundance in *T*.* septentrionalis*. Similarly, many studies have suggested that captivity has a significant impact on the gut microbial structure and abundance from the phylum to genus levels among various vertebrate species, such as reptiles (Jiang et al., 2017), birds (Wang, Cao, et al., 2016; Wang, Zheng, et al., 2016), and mammals (Hale et al., 2018; Mckenzie et al., 2017; Schmidt et al., 2019). Nonetheless, more future experiments are warranted to understand the causes of these changes.

In this study, PICRUSt was also employed to predict the potential gene profiles from 16S rRNA gene sequencing, which allowed for the identification of several functional KEGG categories and pathways expressed in *T*.* septentrionalis*. Our results demonstrated that the most functionally distinct categories were those associated with metabolism, environmental information processing, genetic information processing, cellular processes, human diseases, and organismal systems. Interestingly, although there were no significant differences in the abundances at level1 of the KEGG pathway, gene function predictions showed that at all KEGG pathway levels2 wild lizards had higher gene abundances than did captive lizards, except for genes associated with the circulatory system function (Table A2; Figure 4). In mammals, the bacterial genomes are closely correlated with their natural environments, especially for the host diets (Muegge et al., 2011). Thus, it may be possibly suggested that captivity has a more profound effect on the functional pathways of gut microbes in lizards, and more studies are needed to elucidate the underlying variation mechanism of functional pathways.

## 5 CONCLUSIONS

Our study reveals that the intestinal microbial community composition, abundance, and functional pathways differ between wild and captive northern grass lizards, and such differences may be probably related to changes in the microenvironments (e.g., artificial facilities, without predators) undergone and food ingested by captive lizards. However, the contribution of specific causes needs to be further verified by controlling more environmental factors in future experiments. There are significant differences in the gut microbial community composition and abundance from the class to family levels in *T*.* septentrionalis*. The abundances of almost all KEGG gene pathways levels2 in the gut microbiota are higher in wild than in captive lizard, indicating that the mad‐made environments are often not suitable for wild northern grass lizards.

## CONFLICT OF INTEREST

None declared.

## AUTHOR CONTRIBUTIONS

**Jin Zhou:** Conceptualization (equal); investigation (equal); writing – original draft (equal). **Yu‐Tian Zhao:** Formal analysis (equal); investigation (equal); writing – original draft (equal). **Ying‐Yu Dai:** Investigation (equal). **Yi‐Jin Jiang:** Investigation (equal). **Long‐Hui Lin:** Investigation (equal); writing – original draft (equal). **Hong Li:** Investigation (equal); writing – original draft (equal). **Peng Li:** Investigation (equal); writing – review & editing (equal). **Yan‐Fu Qu:** Conceptualization (equal); formal analysis (equal); resources (equal); supervision (equal); writing – original draft (equal); writing – review & editing (equal). **Xiang Ji:** Conceptualization (equal); resources (equal); writing – original draft (equal); writing – review & editing (equal).

## ETHICS STATEMENT

Our experimental procedures complied with the current laws of China for the care and use of experimental animals and were approved by the Animal Research Ethics Committee of Nanjing Normal University (Permit No. AREC 2018‐03‐02).

## Data Availability

All 16S rRNA gene sequences obtained in this study have been deposited in the NCBI Sequence Read Archive under the BioProject accession number PRJNA597659: https://www.ncbi.nlm.nih.gov/bioproject/PRJNA597659.

## Appendix A

**TABLE A1:** The number of operational taxonomic units (OTUs) and different bacterial taxonomic units of each sample for north grass lizard

Samples	OTUs	Phylum	Class	Order	Family	Genus
NJ_11	208	12	22	34	65	114
NJ_12	111	11	17	25	47	74
NJ_13	107	12	17	21	41	65
NJ_14	91	7	13	20	42	48
NJ_21	130	10	18	25	55	73
NJ_22	139	11	22	27	55	86
NJ_23	124	11	21	27	54	75
NJ_24	154	9	18	29	48	83
Total	286	12	24	41	76	127

**TABLE A2:** The mean relative abundance of gut bacterial taxa at functional gene pathway levels in wild and captivity groups and the *t*‐test results

Pathway level1	Pathway level2	Wild	Captivity	*t* values	*p* values
Cellular processes	Transport and catabolism	0.23415	0.26757	4.88012	0.00277
Cellular processes	Cell motility	2.49469	3.32073	4.60366	0.00368
Cellular processes	Cell growth and death	0.55630	0.42058	3.58020	0.01164
Environmental information processing	Signaling molecules and interaction	0.15159	0.13838	4.36199	0.00476
Environmental information processing	Signal transduction	2.81138	3.00350	4.05231	0.00671
Environmental information processing	Membrane transport	17.10073	17.32874	3.31190	0.01617
Genetic information processing	Replication and repair	8.23937	8.59788	5.03147	0.00238
Genetic information processing	Folding, sorting and degradation	2.96052	2.84124	4.84711	0.00286
Genetic information processing	Translation	5.00362	5.15159	4.71119	0.00329
Genetic information processing	Transcription	2.97263	3.28141	2.95169	0.02556
Human diseases	Immune system diseases	0.05005	0.06190	5.32681	0.00178
Human diseases	Cancers	0.15752	0.15389	4.79998	0.00300
Human diseases	Infectious diseases	0.70915	0.71747	4.43994	0.00438
Human diseases	Neurodegenerative diseases	0.23712	0.25917	4.21048	0.00562
Human diseases	Cardiovascular diseases	0.00382	0.00076	4.05340	0.00670
Human diseases	Metabolic diseases	0.13204	0.10969	3.20886	0.01839
Metabolism	Nucleotide metabolism	3.79570	4.09155	5.16396	0.00209
Metabolism	Biosynthesis of other secondary metabolites	0.99228	0.77951	5.01483	0.00242
Metabolism	Enzyme families	2.68218	2.32961	4.82569	0.00292
Metabolism	Metabolism of terpenoids and polyketides	2.03967	1.91544	4.79856	0.00301
Metabolism	Metabolism of cofactors and vitamins	5.55290	4.86749	4.70785	0.00330
Metabolism	Amino acid metabolism	10.05642	10.60224	4.67351	0.00342
Metabolism	Glycan biosynthesis and metabolism	2.50943	2.89898	4.40636	0.00454
Metabolism	Lipid metabolism	3.36062	3.62387	4.38497	0.00464
Metabolism	Metabolism of other amino acids	1.94112	2.05534	4.37699	0.00468
Metabolism	Xenobiotics biodegradation and metabolism	2.69500	2.63665	4.17199	0.00587
Metabolism	Carbohydrate metabolism	11.53190	11.80087	3.95635	0.00748
Metabolism	Energy metabolism	8.26715	6.12005	3.57251	0.01175
Organismal systems	Nervous system	0.09722	0.08313	4.97648	0.00251
Organismal systems	Environmental adaptation	0.12107	0.13984	4.74883	0.00316
Organismal systems	Immune system	0.07032	0.06277	4.10302	0.00634
Organismal systems	Endocrine system	0.34236	0.26170	3.22479	0.01803
Organismal systems	Excretory system	0.04006	0.03632	3.12052	0.02057
Organismal systems	Digestive system	0.07471	0.02583	2.62491	0.03933
Organismal systems	Circulatory system	0.01522	0.01429	2.25164	0.06529

Note

The degrees of freedom in all *t*‐tests are 6.

## References

[mbo31095-bib-0001] Banks, J. C. , Cary, S. C. , & Hogg, I. D. (2009). The phylogeography of Adelie penguin faecal flora. Environmental Microbiology, 11, 577–588. 10.1111/j.1462-2920.2008.01816.x 19040454

[mbo31095-bib-0002] Benson, A. K. , Kelly, S. A. , Legge, R. , Ma, F. , Low, S. J. , Kim, J. , … Pomp, D. (2010). Individuality in gut microbiota composition is a complex polygenic trait shaped by multiple environmental and host genetic factors. Proceedings of the National Academy of Sciences of the United States of America, 107, 18933–18938. 10.1073/pnas.1007028107 20937875 PMC2973891

[mbo31095-bib-0003] Berry, D. , Schwab, C. , Milinovich, G. , Reichert, J. , Ben Mahfoudh, K. , Decker, T. , … Loy, A. (2012). Phylotype‐level 16S rRNA analysis reveals new bacterial indicators of health state in acute murine colitis. The ISME Journal, 6, 2091–2106. 10.1038/ismej.2012.39 22572638 PMC3475367

[mbo31095-bib-0004] Blaut, M. , Collins, M. D. , Welling, G. W. , Doré, J. , van Loo, J. , & de Vos, W. (2002). Molecular biological methods for studying the gut microbiota: The EU human gut flora project. British Journal of Nutrition, 87, S203–S211. 10.1079/BJN/2002539 12088520

[mbo31095-bib-0005] Boulange, C. L. , Neves, A. L. , Chilloux, J. , Nicholson, J. K. , & Dumas, M. E. (2016). Impact of the gut microbiota on inflammation, obesity, and metabolic disease. Genome Medicine, 8, 42 10.1186/s13073-016-0303-2 27098727 PMC4839080

[mbo31095-bib-0006] Cai, Y. , Yan, J. , Xu, X.‐F. , Lin, Z.‐H. , & Ji, X. (2012). Mitochondrial DNA phylogeography reveals a west‐east division of the northern grass lizard (*Takydromus septentrionalis*) endemic to China. Journal of Zoological Systematics and Evolutionary Research, 50(2), 137–144. 10.1111/j.1439-0469.2012.00655.x

[mbo31095-bib-0007] Campos, P. , Guivernau, M. , Prenafeta‐Boldú, F. X. , & Cardona, L. (2018). Fast acquisition of a polysaccharide fermenting gut microbiome by juvenile green turtles *Chelonia mydas* after settlement in coastal habitats. Microbiome, 6, 69 10.1186/s40168-018-0454-z 29636094 PMC5894180

[mbo31095-bib-0008] Cani, P. D. (2016). Gut microbiota changes in gut microbes and host metabolism: Squaring the circle? Nature Reviews Gastroenterology & Hepatology, 13, 563–564. 10.1038/nrgastro.2016.135 27580685

[mbo31095-bib-0009] Colston, T. J. (2017). Gut microbiome transmission in lizards. Molecular Ecology, 26, 972–974. 10.1111/mec.13987 28239927

[mbo31095-bib-0010] Colston, T. J. , & Jackson, C. R. (2016). Microbiome evolution along divergent branches of the vertebrate tree of life: What is known and unknown. Molecular Ecology, 25, 3776–3800. 10.1111/mec.13730 27297628

[mbo31095-bib-0011] Delport, T. C. , Power, M. L. , Harcourt, R. G. , Webster, K. N. , & Tetu, S. G. (2016). Colony location and captivity influence the gut microbial community composition of the Australian sea lion (*Neophoca cinerea*). Applied and Environmental Microbiology, 82, 3440–3449. 10.1128/AEM.00192-16 27037116 PMC4959163

[mbo31095-bib-0012] Delsuc, F. , Metcalf, J. L. , Parfrey, L. W. , Song, S. J. , González, A. , & Knight, R. (2014). Convergence of gut microbiomes in myrmecophagous mammals. Molecular Ecology, 23, 1301–1317. 10.1111/mec.12501 24118574

[mbo31095-bib-0013] Dewar, M. L. , Arnould, J. P. Y. , Krause, L. , Dann, P. , & Smith, S. C. (2014). Interspecific variations in the faecal microbiota of *Procellariiform* seabirds. FEMS Microbiology Ecology, 89, 47–55. 10.1111/1574-6941.12332 24684257

[mbo31095-bib-0014] Dimitriu, P. A. , Boyce, G. , Samarakoon, A. , Hartmann, M. , Johnson, P. , & Mohn, W. W. (2013). Temporal stability of the mouse gut microbiota in relation to innate and adaptive immunity. Environmental Microbiology Reports, 5, 200–210. 10.1111/j.1758-2229.2012.00393.x 23584963

[mbo31095-bib-0015] Do, T. H. , Nguyen, T. T. , Nguyen, T. N. , Le, Q. G. , Nguyen, C. , Kimura, K. , & Truong, N. H. (2014). Mining biomass‐degrading genes through Illumina‐based de novo sequencing and metagenomic analysis of free‐living bacteria in the gut of the lower termite *Coptotermes gestroi* harvested in Vietnam. Journal of Bioscience and Bioengineering, 118, 665–671. 10.1016/j.jbiosc.2014.05.010 24928651

[mbo31095-bib-0016] Du, W.‐G. , Ji, X. , Zhang, Y.‐P. , Lin, Z.‐H. , & Xu, X.‐F. (2010). Geographic variation in offspring size of a widespread lizard (*Takydromus septentrionalis*): Importance of maternal investment. Biological Journal of the Linnean Society, 101, 59–67. 10.1111/j.1095-8312.2010.01492.x

[mbo31095-bib-0017] Du, W.‐G. , Ji, X. , Zhang, Y.‐P. , Xu, X.‐F. , & Shine, R. (2005). Identifying sources of variation in reproductive and life‐history traits among five populations of a Chinese lizard (*Takydromus septentrionalis*, Lacertidae). Biological Journal of the Linnean Society, 85, 443–453. 10.1111/j.1095-8312.2005.00508.x

[mbo31095-bib-0018] Duncan, S. H. , Lobley, G. E. , Holtrop, G. , Ince, J. , Johnstone, A. M. , Louis, P. , & Flint, H. J. (2008). Human colonic microbiota associated with diet, obesity and weight loss. International Journal of Obesity, 32, 1720–1724. 10.1038/ijo.2008.155 18779823

[mbo31095-bib-0019] Edgar, R. C. (2010). Search and clustering orders of magnitude faster than BLAST. Bioinformatics, 26, 2460–2461. 10.1093/bioinformatics/btq461 20709691

[mbo31095-bib-0020] Edgar, R. C. , Haas, B. J. , Clemente, J. C. , Quince, C. , & Knight, R. (2011). UCHIME improves sensitivity and speed of chimera detection. Bioinformatics, 27, 2194–2200. 10.1093/bioinformatics/btr381 21700674 PMC3150044

[mbo31095-bib-0021] Feldman, A. , Sabath, N. , Pyron, R. A. , Mayrose, I. , & Meiri, S. (2016). Body sizes and diversification rates of lizards, snakes, amphisbaenians and the tuatara. Global Ecology and Biogeography, 25, 187–197. 10.1111/geb.12398

[mbo31095-bib-0022] Flint, H. J. , Scott, K. P. , Duncan, S. H. , Louis, P. , & Forano, E. (2012). Microbial degradation of complex carbohydrates in the gut. Gut Microbes, 3, 289–306. 10.4161/gmic.19897 22572875 PMC3463488

[mbo31095-bib-0023] Greer, R. L. , Dong, X. , Moraes, A. C. F. , Zielke, R. A. , Fernandes, G. R. , Peremyslova, E. , … Shulzhenko, N. (2016). *Akkermansia muciniphila* mediates negative effects of IFNγon glucose metabolism. Nature Communications, 7, 13329 10.1038/ncomms13329 PMC5114536 27841267

[mbo31095-bib-0024] Gupta, A. K. , Nayduch, D. , Verma, P. , Shah, B. , Ghate, H. V. , Patole, M. S. , & Shouche, Y. S. (2012). Phylogenetic characterization of bacteria in the gut of house flies (*Musca domestica* L.). FEMS Microbiology Ecology, 79, 581–593. 10.1111/j.1574-6941.2011.01248.x 22092755

[mbo31095-bib-0025] Hale, V. L. , Tan, C. L. , Niu, K. , Yang, Y. , Knight, R. , Zhang, Q. , … Amato, K. R. (2018). Diet versus phylogeny: A comparison of gut microbiota in captive Colobine monkey species. Microbial Ecology, 75, 515–527. 10.1007/s00248-017-1041-8 28735426

[mbo31095-bib-0026] Heijtz, R. D. , Wang, S. , Anuar, F. , Qian, Y. , Bjorkholm, B. , Samuelsson, A. , … Pettersson, S. (2011). Normal gut microbiota modulates brain development and behavior. Proceedings of the National Academy of Sciences of the United States of America, 108, 3047–3052. 10.1073/pnas.1010529108 21282636 PMC3041077

[mbo31095-bib-0027] Hird, S. M. (2017). Evolutionary biology needs wild microbiomes. Frontiers in Microbiology, 8, 725 10.3389/fmicb.2017.00725 28487687 PMC5404107

[mbo31095-bib-0028] Hird, S. M. , Sánchez, C. , Carstens, B. C. , & Brumfield, R. T. (2015). Comparative gut microbiota of 59 neotropical bird species. Frontiers in Microbiology, 6, 1403 10.3389/fmicb.2015.01403 26733954 PMC4685052

[mbo31095-bib-0029] Hong, P.‐Y. , Wheeler, E. , Cann, I. K. O. , & Mackie, R. I. (2011). Phylogenetic analysis of the fecal microbial community in herbivorous land and marine iguanas of the Galapagos Islands using 16S rRNA‐based pyrosequencing. The ISME Journal, 5, 1461–1470. 10.1038/ismej.2011.33 21451584 PMC3160690

[mbo31095-bib-0030] Hyde, E. R. , Navas‐Molina, J. A. , Song, S. J. , Kueneman, J. G. , Ackermann, G. , Cardona, C. , … Knight, R. (2016). The oral and skin microbiomes of captive komodo dragons are significantly shared with their habitat. mSystems, 1, e00046‐16 10.1128/mSystems.00046-16 27822543 PMC5069958

[mbo31095-bib-0031] Ji, X. , & Diong, C.‐H. (2006). Does follicle excision always result in enlargement of offspring size in lizards? Journal of Comparative Physiology B, 176, 521–525. 10.1007/s00360-006-0074-y 16508736

[mbo31095-bib-0032] Ji, X. , Du, W.‐G. , Lin, Z.‐H. , & Luo, L.‐G. (2007). Measuring temporal variation in reproductive output reveals optimal resource allocation to reproduction in the northern grass lizard, *Takydromus septentrionalis* . Biological Journal of the Linnean Society, 91, 315–324. 10.1111/j.1095-8312.2007.00791.x

[mbo31095-bib-0033] Ji, X. , Du, W.‐G. , & Sun, P.‐Y. (1996). Body temperature, thermal tolerance and influence of temperature on sprint speed and food assimilation in adult grass lizards, *Takydromus septentrionalis* . Journal of Thermal Biology, 21, 155–161. 10.1016/0306-4565(95)00037-2

[mbo31095-bib-0034] Jiang, H.‐Y. , Ma, J.‐E. , Li, J. , Zhang, X.‐J. , Li, L.‐M. , He, N. , … Chen, J.‐P. (2017). Diets alter the gut microbiome of crocodile lizards. Frontiers in Microbiology, 8, 2073 10.3389/fmicb.2017.02073 29118742 PMC5660983

[mbo31095-bib-0035] Kanehisa, M. (2019). Toward understanding the origin and evolution of cellular organisms. Protein Science, 28, 1947–1951. 10.1002/pro.3715 31441146 PMC6798127

[mbo31095-bib-0036] Kartzinel, T. R. , Hsing, J. C. , Musili, P. M. , Brown, B. R. P. , & Pringle, R. M. (2019). Covariation of diet and gut microbiome in African megafauna. Proceedngs of the National Academy of Sciences of the United States of America, 116, 23588–23593. 10.1073/pnas.1905666116 PMC6876249 31685619

[mbo31095-bib-0037] Kohl, K. D. , Brun, A. , Magallanes, M. , Brinkerhoff, J. , Laspiur, A. , Acosta, J. C. , … Bordenstein, S. R. (2017). Gut microbial ecology of lizards: Insights into diversity in the wild, effects of captivity, variation across gut regions and transmission. Molecular Ecology, 26, 1175–1189. 10.1111/mec.13921 27862531

[mbo31095-bib-0038] Kohl, K. D. , Sadowska, E. T. , Rudolf, A. M. , Dearing, M. D. , & Koteja, P. (2016). Experimental evolution on a wild mammal species results in modifications of gut microbial communities. Frontiers in Microbiology, 7, 634 10.3389/fmicb.7016.00634 27199960 PMC4854874

[mbo31095-bib-0039] Kohl, K. D. , Skopec, M. M. , & Dearing, M. D. (2014). Captivity results in disparate loss of gut microbial diversity in closely related hosts. Conservation Physiology, 2, cou009 10.1093/conphys/cou009 27293630 PMC4806740

[mbo31095-bib-0040] Kormas, K. A. , Meziti, A. , Mente, E. , & Frentzos, A. (2014). Dietary differences are reflected on the gut prokaryotic community structure of wild and commercially reared sea bream (*Sparus aurata*). Microbiology Open, 3, 718–728. 10.1002/mbo3.202 25066034 PMC4234263

[mbo31095-bib-0041] Langille, M. G. I. , Zaneveld, J. , Caporaso, J. G. , McDonald, D. , Knights, D. , Reyes, J. A. , … Huttenhower, C. (2013). Predictive functional profiling of microbial communities using 16S rRNA marker gene sequences. Nature Biotechnology, 31, 814–821. 10.1038/nbt.2676 PMC3819121 23975157

[mbo31095-bib-0042] Leftwich, P. T. , Clarke, N. V. E. , Hutchings, M. I. , & Chapman, T. (2017). Gut microbiomes and reproductive isolation in *Drosophila* . Proceedings of the National Academy of Sciences of the United States of America, 114, 12767–12772. 10.1073/pnas.1708345114 29109277 PMC5715749

[mbo31095-bib-0043] Lewin, W.‐C. , Kamjunke, N. , & Mehner, T. (2003). Phosphorus uptake by microcystis during passage through fish guts. Limnology and Oceanography, 48, 2392–2396. 10.4319/lo.2003.48.6.2392

[mbo31095-bib-0044] Li, H. , Li, T. , Beasley, D. A. E. , Heděnec, P. , Xiao, Z. , Zhang, S. , … Li, X. (2016). Diet diversity is associated with beta but not alpha diversity of pika gut microbiota. Frontiers in Microbiology, 7, 1169 10.3389/fmicb.2016.01169 27512391 PMC4961685

[mbo31095-bib-0045] Li, X.‐M. , Zhu, Y.‐J. , Yan, Q.‐Y. , Ringø, E. , & Yang, D.‐G. (2014). Do the intestinal microbiotas differ between paddlefish (*Polyodon spathala*) and bighead carp (*Aristichthys nobilis*) reared in the same pond? Journal of Applied Microbiology, 117, 1245–1252. 10.1111/jam.12626 25155438

[mbo31095-bib-0046] Lindheim, L. , Bashir, M. , Münzker, J. , Trummer, C. , Zachhuber, V. , Leber, B. , … Obermayer‐Pietsch, B. (2017). Alterations in gut microbiome composition and barrier function are associated with reproductive and metabolic defects in women with polycystic ovary syndrome (PCOS): A pilot study. PLoS One, 12, e0168390 10.1371/journal.pone.0168390 28045919 PMC5207627

[mbo31095-bib-0047] Liu, M.‐Y. (1999). *Takydromus septentrionalis* (Günther, 1864) In Zhao E. M., Zhao K. T., & Zhou K. Y. (Eds.), Fauna Sinica, Reptilia (Squamata: Lacertilia) (Vol. 2, pp. 263–266). Beijing, China: Science Press.

[mbo31095-bib-0048] Luo, L.‐G. , Ding, G.‐H. , & Ji, X. (2010). Income breeding and temperature‐induced plasticity in reproductive traits in lizards. Journal of Experimental Biology, 213, 2073–2078. 10.1242/jeb.041137 20511521

[mbo31095-bib-0049] Magne, F. , Abely, M. , Boyer, F. , Morville, P. , Pochart, P. , & Suau, A. (2006). Low species diversity and high interindividual variability in faeces of preterm infants as revealed by sequences of 16S rRNA genes and PCR‐temporal temperature gradient gel electrophoresis profiles. FEMS Microbiology Ecology, 57, 128–138. 10.1111/j.1574-6941.2006.00097.x 16819956

[mbo31095-bib-0050] Martin, M. O. , Gilman, F. R. , & Weiss, S. L. (2010). Sex‐specific asymmetry within the cloacal microbiota of the striped plateau lizard, *Sceloporus virgatus* . Symbiosis, 51, 97–105. 10.1007/s13199-010-0078-y

[mbo31095-bib-0051] Matsen, F. A. (2015). Phylogenetics and the human microbiome. Systematic Biology, 64, E26–E41. 10.1093/sysbio/syu053 25102857 PMC4265140

[mbo31095-bib-0052] McKenzie, V. J. , Song, S. J. , Delsuc, F. , Prest, T. L. , Oliverio, A. M. , Korpita, T. M. , … Knight, R. (2017). The effects of captivity on the mammalian gut microbiome. Integrative and Comparative Biology, 57, 690–704. 10.1093/icb/icx090 28985326 PMC5978021

[mbo31095-bib-0053] Montalban‐Arques, A. , De Schryver, P. , Bossier, P. , Gorkiewicz, G. , Mulero, V. , Gatlin, D. M. , & Galindo‐Villegas, J. (2015). Selective manipulation of the gut microbiota improves immune status in vertebrates. Frontiers in Immunology, 6, 512 10.3389/fimmu.2015.00512 26500650 PMC4598590

[mbo31095-bib-0054] Muegge, B. D. , Kuczynski, J. , Knights, D. , Clemente, J. C. , Gonzalez, A. , Fontana, L. , … Gordon, J. I. (2011). Diet drives convergence in gut microbiome functions across mammalian phylogeny and within humans. Science, 332, 970–974. 10.1126/science.1198719 21596990 PMC3303602

[mbo31095-bib-0055] Nakamura, N. , Amato, K. R. , Garber, P. , Estrada, A. , Mackie, R. I. , & Gaskins, H. R. (2011). Analysis of the hydrogenotrophic microbiota of wild and captive black howler monkeys (*Alouatta pigra*) in palenque national park, Mexico. AmericanJournal of Primatology, 73, 909–919. 10.1002/ajp.20961 21557284

[mbo31095-bib-0056] Nechitaylo, T. Y. , Yakimov, M. M. , Godinho, M. , Timmis, K. N. , Belogolova, E. , Byzov, B. A. , … Golyshin, P. N. (2010). Effect of the earthworms *Lumbricus terrestris* and *Aporrectodea caliginosa* on bacterial diversity in soil. Microbial Ecology, 59, 574–587. 10.1007/s00248-009-9604-y 19888626

[mbo31095-bib-0057] Nelson, D. M. , Cann, I. K. O. , Altermann, E. , & Mackie, R. I. (2010). Phylogenetic evidence for lateral gene transfer in the intestine of marine iguanas. PLoS One, 5, e10785 10.1371/journal.pone.0010785 20520734 PMC2875401

[mbo31095-bib-0058] Nelson, T. M. , Rogers, T. L. , Carlini, A. R. , & Brown, M. V. (2013). Diet and phylogeny shape the gut microbiota of Antarctic seals: A comparison of wild and captive animals. Environmental Microbiology, 15, 1132–1145. 10.1111/1462-2920.12022 23145888

[mbo31095-bib-0059] Ng, S. H. , Stat, M. , Bunce, M. , & Simmons, L. W. (2018). The influence of diet and environment on the gut microbial community of field crickets. Ecology and Evolution, 8, 4704–4720. 10.1002/ece3.3977 29760910 PMC5938447

[mbo31095-bib-0060] Oksanen, J. , Blanchet, F. G. , Kindt, R. , Legendre, P. , Minchin, P. R. , O'Hara, R. B. , … Wagner, H. H. (2013). Vegan: Community ecology package. R package version 2.0‐10.

[mbo31095-bib-0061] Opstelten, J. L. , Plassais, J. , van Mil, S. W. C. , Achouri, E. , Pichaud, M. , Siersema, P. D. , … Cervino, A. C. L. (2016). Gut microbial diversity is reduced in smokers with Crohn's disease. Inflammatory Bowel Diseases, 22, 2070–2077. 10.1097/mib.0000000000000875 27542127 PMC4991341

[mbo31095-bib-0062] Org, E. , Parks, B. W. , Joo, J. W. J. , Emert, B. , Schwartzman, W. , Kang, E. Y. , … Lusis, A. J. (2015). Genetic and environmental control of host‐gut microbiota interactions. Genome Research, 25, 1558–1569. 10.1101/gr.194118.115 26260972 PMC4579341

[mbo31095-bib-0063] Price, J. T. , Paladino, F. V. , Lamont, M. M. , Witherington, B. E. , Bates, S. T. , & Soule, T. (2017). Characterization of the juvenile green turtle (*Chelonia mydas*) microbiome throughout an ontogenetic shift from pelagic to neritic habitats. PLoS One, 12, e0177642 10.1371/journal.pone.0177642 28493980 PMC5426784

[mbo31095-bib-0064] R Development Core Team (2019). R: A language and environment for statistical computing. Vienna, Austria: R Foundation For Statistical Computing Retrieved from http://www.R‐project.org

[mbo31095-bib-0065] Reid, N. M. , Addison, S. L. , Macdonald, L. J. , & Lloyd‐Jones, G. (2011). Biodiversity of active and inactive bacteria in the gut flora of wood‐feeding huhu beetle larvae (*Prionoplus reticularis*). Applied and Environmental Microbiology, 77, 7000–7006. 10.1128/AEM.05609-11 21841025 PMC3187079

[mbo31095-bib-0066] Ren, T. , Kahrl, A. F. , Wu, M. , & Cox, R. M. (2016). Does adaptive radiation of a host lineage promote ecological diversity of its bacterial communities? A test using gut microbiota of *Anolis* lizards. Molecular Ecology, 25, 4793–4804. 10.1111/mec.13796 27497270

[mbo31095-bib-0067] Rowland, I. , Gibson, G. , Heinken, A. , Scott, K. , Swann, J. , Thiele, I. , & Tuohy, K. (2018). Gut microbiota functions: Metabolism of nutrients and other food components. European Journal of Nutrition, 57, 1–24. 10.1007/s00394-017-1445-8 PMC5847071 28393285

[mbo31095-bib-0068] Rungrassamee, W. , Klanchui, A. , Maibunkaew, S. , Chaiyapechara, S. , Jiravanichpaisal, P. , & Karoonuthaisiri, N. (2014). Characterization of intestinal bacteria in wild and domesticated adult black tiger shrimp (*Penaeus monodon*). PLoS One, 9, e91853 10.1371/journal.pone.0091853 24618668 PMC3950284

[mbo31095-bib-0069] Schloss, P. D. , Westcott, S. L. , Ryabin, T. , Hall, J. R. , Hartmann, M. , Hollister, E. B. , … Weber, C. F. (2009). Introducing mothur: Open‐source, platform‐independent, community‐supported software for describing and comparing microbial communities. Applied and Environmental Microbiology, 75, 7537–7541. 10.1128/AEM.01541-09 19801464 PMC2786419

[mbo31095-bib-0070] Schmidt, E. , Mykytczuk, N. C. S. , & Schultehostedde, A. I. (2019). Effects of the captive and wild environment on diversity of the gut microbiome of deer mice (*Peromyscus maniculatus*). The ISME Journal, 13, 1293–1305. 10.1038/s41396-019-0345-8 30664674 PMC6474230

[mbo31095-bib-0071] Schmieder, R. , & Edwards, R. (2011). Quality control and preprocessing of metagenomic datasets. Bioinformatics, 27, 863–864. 10.1093/bioinformatics/btr026 21278185 PMC3051327

[mbo31095-bib-0072] Segata, N. , Izard, J. , Waldron, L. , Gevers, D. , Miropolsky, L. , Garrett, W. S. , & Huttenhower, C. (2011). Metagenomic biomarker discovery and explanation. Genome Biology, 12, R60 10.1186/gb-2011-12-6-r60 21702898 PMC3218848

[mbo31095-bib-0073] Semova, I. , Carten, J. D. , Stombaugh, J. , Mackey, L. C. , Knight, R. , Farber, S. A. , & Rawls, J. F. (2012). Microbiota regulate intestinal absorption and metabolism of fatty acids in the zebrafish. Cell Host & Microbe, 12, 277–288. 10.1016/j.chom.2012.08.003 22980325 PMC3517662

[mbo31095-bib-0074] Shine, R. (2005). Life‐history evolution in reptiles. Annual Review of Ecology Evolution and Systematics, 36, 23–46. 10.1146/annurev.ecolsys.36.102003.152631

[mbo31095-bib-0075] Spor, A. , Koren, O. , & Ley, R. (2011). Unravelling the effects of the environment and host genotype on the gut microbiome. Nature Reviews Microbiology, 9, 279–290. 10.1038/nrmicro2540 21407244

[mbo31095-bib-0076] Sullam, K. E. , Essinger, S. D. , Lozupone, C. A. , O'connor, M. P. , Rosen, G. L. , Knight, R. , … Russell, J. A. (2012). Environmental and ecological factors that shape the gut bacterial communities of fish: A meta‐analysis. Molecular Ecology, 21, 3363–3378. 10.1111/j.1365-294X.2012.05552.x 22486918 PMC3882143

[mbo31095-bib-0077] Suzuki, T. A. , & Worobey, M. (2014). Geographical variation of human gut microbial composition. Biology Letters, 10, 20131037 10.1098/rsbl.2013.1037 24522631 PMC3949373

[mbo31095-bib-0078] Tap, J. , Furet, J.‐P. , Bensaada, M. , Philippe, C. , Roth, H. , Rabot, S. , … Leclerc, M. (2015). Gut microbiota richness promotes its stability upon increased dietary fibre intake in healthy adults. Environmental Microbiology, 17, 4954–4964. 10.1111/1462-2920.13006 26235304

[mbo31095-bib-0079] Tsukayama, P. , Boolchandani, M. , Patel, S. , Pehrsson, E. C. , Gibson, M. K. , Chiou, K. L. , … Dantas, G. (2018). Characterization of wild and captive baboon gut microbiota and their antibiotic resistomes. mSystems, 3, e00016–e00018. 10.1128/mSystems.00016-18 29963641 PMC6020475

[mbo31095-bib-0080] Vaz, V. C. , Santori, R. T. , Jansen, A. M. , Delciellos, A. C. , & D'Andrea, P. S. (2012). Notes on food habits of armadillos (Cingulata, Dasypodidae) and anteaters (Pilosa, Myrmecophagidae) at Serra Da Capivara National Park (PiauÍ State, Brazil). Edentata, 13, 84–89. 10.5537/020.013.0107

[mbo31095-bib-0081] Vences, M. , Lyra, M. L. , Kueneman, J. G. , Bletz, M. C. , Archer, H. M. , Canitz, J. , … Glos, J. (2016). Gut bacterial communities across tadpole ecomorphs in two diverse tropical anuran faunas. Science of Nature, 103, 25 10.1007/s00114-016-1348-1 26924012

[mbo31095-bib-0082] Villers, L. M. , Jang, S. S. , Lent, C. L. , Lewin‐Koh, S.‐C. , & Norosoarinaivo, J. A. (2008). Survey and comparison of major intestinal flora in captive and wild ring‐tailed lemur (*Lemur catta*) populations. American Journal of Primatology, 70, 175–184. 10.1002/ajp.20482 17854057

[mbo31095-bib-0083] Wang, W. , Cao, J. , Yang, F. , Wang, X.‐L. , Zheng, S.‐S. , Sharshov, K. , & Li, L.‐X. (2016). High‐throughput sequencing reveals the core gut microbiome of bar‐headed goose (*Anser indicus*) in different wintering areas in Tibet. Microbiology Open, 5, 287–295. 10.1002/mbo3.327 26842811 PMC4831473

[mbo31095-bib-0084] Wang, W. , Zheng, S.‐S. , Sharshov, K. , Cao, J. , Sun, H. , Yang, F. , … Li, L.‐X. (2016). Distinctive gut microbial community structure in both the wild and farmed Swan goose (*Anser cygnoides*). Journal of Basic Microbiology, 56, 1299–1307. 10.1002/jobm.201600155 27365218

[mbo31095-bib-0085] Woltering, J. M. (2012). From lizard to snake; behind the evolution of an extreme body plan. Current Genomics, 13, 289–299. 10.2174/138920212800793302 23204918 PMC3394116

[mbo31095-bib-0086] Wong, S. , Stephens, W. Z. , Burns, A. R. , Stagaman, K. , David, L. A. , Bohannan, B. J. M. , … Rawls, J. F. (2015). Ontogenetic differences in dietary fat influence microbiota assembly in the zebrafish gut. MBio, 6, e00687‐15 10.1128/mBio.00687-15 26419876 PMC4611033

[mbo31095-bib-0087] Yang, J. , Sun, Y.‐Y. , An, H. , & Ji, X. (2008). Northern grass lizards (*Takydromus septentrionalis*) from different populations do not differ in thermal preference and thermal tolerance when acclimated under identical thermal conditions. Journal of Comparative Physiology B, 178, 343–349. 10.1007/s00360-007-0227-7 18071715

[mbo31095-bib-0088] Ye, L. , Amberg, J. , Chapman, D. , Gaikowski, M. , & Liu, W.‐T. (2014). Fish gut microbiota analysis differentiates physiology and behavior of invasive Asian carp and indigenous American fish. The ISME Journal, 8, 541–551. 10.1038/ismej.2013.181 24132079 PMC3930320

[mbo31095-bib-0089] Yun, J.‐H. , Roh, S. W. , Whon, T. W. , Jung, M.‐J. , Kim, M.‐S. , Park, D.‐S. , … Bae, J.‐W. (2014). Insect gut bacterial diversity determined by environmental habitat, diet, developmental stage, and phylogeny of host. Applied and Environmental Microbiology, 80, 5254–5264. 10.1128/AEM.01226-14 24928884 PMC4136111

[mbo31095-bib-0090] Zhang, J.‐J. , Kobert, K. , Flouri, T. , & Stamatakis, A. (2014). PEAR: A fast and accurate Illumina paired‐end reAd mergeR. Bioinformatics, 30, 614–620. 10.1093/bioinformatics/btt593 24142950 PMC3933873

[mbo31095-bib-0091] Zhang, W.‐Y. , Li, N. , Tang, X.‐L. , Liu, N.‐F. , & Zhao, W. (2018). Changes in intestinal microbiota across an altitudinal gradient in the lizard *Phrynocephalus vlangalii* . Ecology and Evolution, 8, 4695–4703. 10.1002/ece3.4029 29760909 PMC5938461

[mbo31095-bib-0092] Zhao, J. , Yao, Y. , Li, D. , Xu, H. , Wu, J. , Wen, A. , … Xu, H. (2018). Characterization of the gut microbiota in six geographical populations of Chinese rhesus macaques (*Macaca mulatta*), implying an adaptation to high‐altitude environment. Microbial Ecology, 76, 565–577. 10.1007/s00248-018-1146-8 29372281

